# Dominance of variant A in Human Herpesvirus 6 viraemia after renal transplantation

**DOI:** 10.1186/1743-422X-8-403

**Published:** 2011-08-15

**Authors:** Eszter Csoma, Beáta Mészáros, Tamás Gáll, László Asztalos, József Kónya, Lajos Gergely

**Affiliations:** 1 Institute of Medical Microbiology, University of Debrecen, Nagyerdei krt. 98., Debrecen, Hungary; 2 Institute of Surgery, University of Debrecen, Nagyerdei krt. 98., Debrecen, Hungary

**Keywords:** HHV-6, variant A, renal transplantation

## Abstract

**Background:**

Human herpesvirus 6 (HHV-6), mostly variant B reactivation in renal transplant patients has been published by other authors, but the pathogenetic role of HHV-6 variant A has not been clarified. Our aims were to examine the prevalence of HHV-6, to determine the variants, and to investigate the interaction between HHV-6 viraemia, human cytomegalovirus (HCMV) infection and clinical symptoms.

**Methods:**

Variant-specific HHV-6 nested PCR and quantitative real-time PCR were used to examine blood samples from renal transplant patients and healthy blood donors for the presence and load of HHV-6 DNA and to determine the variants. Active HHV-6 infection was proved by RT-PCR, and active HCMV infection was diagnosed by pp65 antigenaemia test.

**Results:**

HHV-6 viraemia was significantly more frequent in renal transplant patients compared to healthy blood donors (9/200 vs. 0/200; p = 0.004), while prevalence of HHV-6 latency was not significantly different (13/200 vs. 19/200; p > 0.05). Dominance of variant A was revealed in viraemias (8/9), and the frequency of HHV-6A was significantly higher in active infections compared with latency in renal transplant patients (8/9 vs. 2/13; p = 0.0015). Latency was established predominantly by HHV-6B both in renal transplant patients and in healthy blood donors (11/13 and 18/19). There was no statistical significant difference in occurrence of HCMV and HHV-6 viraemia in renal transplant patients (7/200 vs. 9/200). Statistical analysis did not reveal interaction between HHV-6 viraemia and clinical symptoms in our study.

**Conclusions:**

Contrary to previous publications HHV-6A viraemia was found to be predominant in renal transplant patients. Frequency of variant A was significantly higher in cases of active infection then in latency.

## Background

Immunosuppression associated with renal transplantation presents a risk for opportunistic infections, reactivations and reinfections. Human herpesvirus 6 (HHV-6) is an important pathogen in transplant recipients. HHV-6 is ubiquitous in the population, primary infection occurs in early childhood after which latency is established and the seropositivity exceeds 90% [1]. There are two distinct variants of HHV-6 [2]: variants A and B (HHV-6A, HHV-6B). Primary infection almost always occurs with HHV-6B [3], but it is not clarified when HHV-6A infection takes place. HHV-6 infection in transplant patients may be the result of donor transmission, reactivation of latent infection in the recipients or reinfection [4]. HHV-6 reactivation occurs early, 38-60% of the patients are affected 2-4 weeks after transplantation [5-7], but late infections up to 2 years has been also described [8,9]. Infection is often asymptomatic [10], but reactivations can result in fever, rash, thrombocytopenia, leukopenia, pneumonia, hepatitis, pancreatitis, colitis, encephalitis, meningoencephalitis, even prolonged bone marrow suppression [4]. HHV-6 can also modulate the immune system which can result in spread and persistence of HHV-6 and can enhance the effects of other infection [11]. There is increasing evidence for simultaneous reactivation of HHV-6 and HCMV [12-15], an action which predicts higher risk for severe disease [15]. It is also suggested that HHV-6 reactivation occurs earlier than HCMV [12,14] which may indicate that HHV-6 can play a role in the reactivation of HCMV by induction of immunosuppression or by interaction with HCMV. In peripheral blood samples of renal transplant patients HHV-6 variant B is more frequently isolated [16,17], but variant A may be more virulent [18]. The aims of this study were to investigate the prevalence of HHV-6 infection in renal transplant patients at different times after transplantation, to determine the subtype of HHV-6, and to examine the possible association of HHV-6 infection with HCMV reactivation and clinical symptoms.

## Results

### HHV-6 viraemia

Twelve plasma and white blood cells (WBC) samples (6%) of 12 renal transplant patients were HHV-6 DNA positive. Dominance of HHV-6 variant A was revealed (10/12). The level of HHV-6A DNA in plasma samples ranged from 7.5 × 10^2^ to 6 × 10^5^ (median 5.9 × 10^3^) genome equivalent/mL (GEq/mL), while the copy number of HHV-6B genome was below the limit of detection (less then 250 GEq/mL). In WBC samples HHV-6 DNA load ranged from 5.1 × 10^2 ^to 2.1 × 10^6 ^(median 1.8 × 10^3^) GEq/1.5 x10^6^ cells. Three samples were negative for HHV-6 mRNA, while RT-PCR proved active HHV-6 infection in nine samples (8 HHV-6A and 1 HHV-6B; Table 1).

**Table 1:** Prevalence of HHV-6 and HHV-6 variants A and B in renal transplant patients and healthy controls

	Viraemia		Latency	
	**Transplant**	**Healthy**	**Transplant**	**Healthy**

HHV-6 (all)	9/200	0/200	13/200	19/200

	p = 0.004		p > 0.05	

HHV-6B	1/9	0/0	11/13	18/19

HHV-6A	8/9	0/0	2/13	1/19

	p = 0.0015			

Statistical analysis did not reveal significant age differences between HHV-6 positive (range 17.9-61.7 years; median 37.8 years) and negative (11.2-68.8 years; median 45.4 years) patients. There was also not significant difference between the time of sample taking after the transplantation of HHV-6 positive (range 21 days-13.1 years; median 6.2 years) and negative (range 3 days-19.6 years, median 3.3 years) patients.

Among healthy blood donors HHV-6 DNA, variant A was detected in both the plasma and WBC of one sample; 5.8 × 10^3^ GEq/1.5 × 10^6^ WBC was measured, but the viral load in plasma was below the limit of detection. RT-PCR did not confirm active infection (Table 1).

### HHV-6 latency

HHV-6 latency was revealed in 13 renal transplant patients (6.5%; 11 HHV-6B and 2 HHV-6A; Table 1). HHV-6 DNA was detected only in WBC. HHV-6 mRNA was not found in these samples.

HHV-6 DNA was found in WBC without active HHV-6 replication in 19 healthy blood donors (9.5%; 18 HHV-6B and 1 HHV-6A; Table 1).

### HCMV reactivation

Seven patients (3.5%) had HCMV reactivation shown by detection of pp65 antigen in WBC.

Statistical analysis did not show significant age differences between HCMV positive (range 44.1-61.6 years; median 55.4 years) and negative (11.2-65.7 years; median 44.8 years) patients. There was also not significant difference between the time of sample taking after the transplantation of HCMV positive (range 50 days-15.7 years; median 0.3 years) and negative (range 21 days-13.1 years, median 6 years) patients.

Simultaneous presence of HCMV and HHV-6 viraemia was not detected.

### Clinical data

Thirty one patients did not have clinical symptoms; fever, respiratory and gastrointestinal symptoms were observed in 169 patients (Table 2). All the patients had good graft functions at the time of the examination.

**Table 2:** Clinical data of 200 renal transplant patients

	Number of patients			
	
	HHV-6+	HHV-6-	HCMV+	HCMV-
**Respiratory symptoms**	2	78	3	77

**Respiratory symptoms with fever**	5	53	1	57

**Respiratory and gastrointestinal symptoms**	1	1	0	2

**Gastrointestinal symptoms**	1	10	2	9

**Gastrointestinal symptoms with fever**	0	6	0	6

**Fever**	0	12	0	12

**No clinical symptoms**	0	31	1	30

Total	9	191	7	193

Statistical analyses of HHV-6 viraemia and clinical symptoms did not reveal association between the presence of virus and the observed clinical symptoms.

## Discussion

In this study significantly higher prevalence of HHV-6 viraemia was found among renal transplant patients then among healthy blood donors (9/200 vs. 0/200; p = 0.004). The frequency of HHV-6 infection was not significantly different from the frequency of HCMV reactivation (9/200 vs. 7/200; p > 0.05). Previous reports have noted that HHV-6 viraemia was found early, but also late after transplantation [4,13,14]. Significant difference was not found between HHV-6 positive and negative patients regarding the days after transplantation at the time of sample collection (p > 0.05). HHV-6 is able to establish latency and integrate into human chromosomes, hence viral DNA can originate from lysis of cells. The incidence of chromosomally integrated HHV-6 (CIHHV-6) is about 0.2-3% [19-21]. Individuals with CIHHV-6 have significant viral load, over million of GEq/mL blood; infection or reactivation usually results in only tens of thousands GEq/mL. It is also suggested that finding of variant A indicates CIHHV-6 rather then active infection [20,22]. CIHHV-6 can also be reactivated and cells harbouring CIHHV-6 will produce infectious viral particles [23,24]. In this study, the viral load detected in the blood samples of the renal transplant patients were in accordance with average DNA load of active HHV-6 infections.

Contrary to some previous publications [17,25-27], HHV-6A viraemia was found to be more frequent (eight out of nine HHV-6 positive samples) in renal transplant individuals then HHV-6 variant B. Nowadays, effective immunosuppressive protocols with new drugs result in improved survival of grafts in organ transplant patients. However, owing to the strong immunosuppression infection and consequently hospitalization is increasing among renal transplant patients [4]. Nearly all of the symptomatic primary HHV-6 infections in children are caused by variant B, and HHV-6B is detected predominantly in healthy individuals. HHV-6A has been identified rarely, but often in severe inflammatory and neurological diseases especially in immunosuppressed patients [28]. HHV-6A dominance was observed in human immunodeficiency virus infected individuals, and it may foster the progression of AIDS [11]. HHV-6A may be an emerging pathogen, and strong immunosuppression of transplant patients might result in higher frequency of HHV-6A infection. In our study, the prevalence of latent HHV-6 infection was not significantly different between renal transplant patients and healthy blood donors (13/200 vs. 19/200; p > 0.05). Dominance of variant B was observed in transplant patients (11/13) and also in healthy blood donors (18/19 latency). The frequency of variant A was significantly higher in viraemia then in latency in renal transplant patients (8/9 vs. 2/13; p = 0.0015). HHV-6 variant A can result from latency, primary infection or reinfection.

Statistical analysis did not find interaction between HHV-6 viraemia and clinical symptoms in our study. All of the patients with HHV-6 viraemia had mild diseases at the time of sample taking, but most of the patients without HHV-6 infection or with latency also had (9/9 vs. 160/191; p > 0.05). Nevertheless, it was not a follow up study, and we have no data before and after the observed HHV-6 viraemia.

HHV-6 and HCMV viraemia was not observed simultaneously in renal transplant patients, which does not exclude the interaction between these viruses.

## Conclusions

In this study HHV-6 viraemia was detected early and late after renal transplantation. In contrast to previous reports, HHV-6 variant A viraemia was found to be predominant in these patients. Frequency of variant A was significantly higher in immunocompromised patients with active infection then with latency. A follow up study of renal transplant patients may reveal the clinical importance of HHV-6A and its potential pathogenetical role.

## Methods

### Patients and samples

Two hundred EDTA blood samples from 200 renal transplant patients (114 men, 86 women, median age 45.5, range 11.2-68.8 years) were taken at different times after transplantation (median 1271 days, range 3-7115 days). The pre-transplant HHV-6 serostatus of the patients was not determined. Calcineurin inhibitor, steroid and mycophenolate mofetil was used according to standard immunosuppressive protocols of patients.

Two hundred EDTA blood samples from 200 healthy blood donors were also collected (75 men, 125 women, median age 39, range 10-74 years).

Regional and Institutional Ethics Committee of University of Debrecen approved all of the studies. All patients gave their written informed consent.

### Qualitative and quantitative PCR of HHV-6

Nucleic acid from white blood cells (WBC) (1.5 × 10^6 ^cells/blood sample) and 200 μL plasma (centrifuged for 10 min at 180 × *g *at 4°C) was isolated by High Pure Viral Nucleic Acid Kit (Roche, Switzerland) according to the manufacturer's instructions. Nucleic acid was eluted in 50 μL and stored at -20°C until usage.

Variant-specific nested PCR amplification of HHV-6 DNA was performed in a final volume of 20 μL containing 5 μL DNA solutions as described previously [29].

To distinguish between latent and active HHV-6 infection, reverse transcription PCR (RT-PCR) was used as described previously [30].

The effectiveness of DNA and RNA isolation were controlled by use of PCR and RT-PCR amplification of β-globin DNA and GAPDH mRNA.

Absolute quantification of HHV-6 DNA using real-time PCR was performed following the method of Boutolleau et al [31]. For calibration curve plasmid DNA (pGL2-Basic vector, Promega, USA) containing HHV-6 insert was used.

### HCMV pp65 antigenaemia

HCMV reactivation was examined by pp65-antigenaemia using CINAkit (Argene, France) according to the manufacturer's instructions.

### Statistical analysis

Chi sqare test and Fisher's exact test were used to asses the difference in frequency for categorical variables. Mann-Whitney U test was applied for continuous variables. Difference was considered significant if p value was less then 0.05.

## Competing interests

The authors declare that they have no competing interests.

## Authors' contributions

ECs contributed to the conception and design of the study and acquisition of funding, carried out the sample collection, nucleic acid isolation, nested PCR, RT-PCR, viral load assays and drafted the manuscript. BM contributed to the sample collection, nucleic acid isolation, nested PCR, RT-PCR. AL provided the clinical samples and clinical data. TG contributed to positive controls and plasmid production. JK performed statistical analysis. LG contributed to the acquisition of funding and revised the manuscript. All authors read and approved the final manuscript.
